# Beyond Face Value: Does Involuntary Emotional Anticipation Shape the Perception of Dynamic Facial Expressions?

**DOI:** 10.1371/journal.pone.0056003

**Published:** 2013-02-11

**Authors:** Letizia Palumbo, Tjeerd Jellema

**Affiliations:** Department of Psychology, University of Hull, Hull, United Kingdom; Royal Holloway, University of London, United Kingdom

## Abstract

Emotional facial expressions are immediate indicators of the affective dispositions of others. Recently it has been shown that early stages of social perception can already be influenced by (implicit) attributions made by the observer about the agent’s mental state and intentions. In the current study possible mechanisms underpinning distortions in the perception of dynamic, ecologically-valid, facial expressions were explored. In four experiments we examined to what extent basic perceptual processes such as contrast/context effects, adaptation and representational momentum underpinned the perceptual distortions, and to what extent ‘emotional anticipation’, i.e. the involuntary anticipation of the other’s emotional state of mind on the basis of the immediate perceptual history, might have played a role. Neutral facial expressions displayed at the end of short video-clips, in which an initial facial expression of joy or anger gradually morphed into a neutral expression, were misjudged as being slightly angry or slightly happy, respectively (Experiment 1). This response bias disappeared when the actor’s identity changed in the final neutral expression (Experiment 2). Videos depicting neutral-to-joy-to-neutral and neutral-to-anger-to-neutral sequences again produced biases but in opposite direction (Experiment 3). The bias survived insertion of a 400 ms blank (Experiment 4). These results suggested that the perceptual distortions were not caused by any of the low-level perceptual mechanisms (adaptation, representational momentum and contrast effects). We speculate that especially when presented with dynamic, facial expressions, perceptual distortions occur that reflect ‘emotional anticipation’ (a low-level mindreading mechanism), which overrules low-level visual mechanisms. Underpinning neural mechanisms are discussed in relation to the current debate on action and emotion understanding.

## Introduction

It is virtually impossible to look at an expression displayed on a human face and not get an immediate impression of the individual’s emotional state of mind. It is as if we directly perceive the emotional/mental state *in* the facial expression, a point emphasised by the phenomenological tradition in philosophy of mind [1]. Wittgenstein, although not a phenomenologist himself, remarked: “We *see* emotion. - As opposed to what? - We do not see facial contortions and *make the inference* that he is feeling joy, grief, boredom. We describe a face immediately as sad, radiant, bored, even when we are unable to give any other description of the features. - Grief, one would like to say, is personified in the face.” [2]. These ideas have been reinvigorated by contemporary philosophers [3] and are echoed by a renewed interest into the interplay between bottom-up visual processes and top-down attributions of mental/emotional states [4].

The processing of social stimuli, such as emotional facial expressions, is nevertheless typically considered to be essentially a bottom-up process. That is, initially there is a perceptual categorization of the facial expression on the basis of its physical features, which subsequently, in combination with contextual information, is used to infer the emotional/mental dispositions of the agent [5]. However, recent theories of the embodiment of others’ facial expressions [6]–[10] have suggested that there might also be an influence which works in the other way: the perceptual categorization process could actually be induced (or facilitated) by the involuntary ‘experiential’ appreciation of the agent’s emotional/mental state. In this latter view, an involuntary motor simulation of the observed action, enabled by mirror mechanisms [11], [12], causes the observer to ‘experience’ the observed action, which informs about the agent’s emotional/mental state and influences the perceptual categorization of the action [13]. The motor simulation further generates an expectation/anticipation of the most likely next action and accompanying mental state [14]. Where deliberate inferences and contextual knowledge can be said to provide a third person perspective, the embodiment (motor simulation) provides the observer with an ‘experiential’ understanding from a first person perspective; these two perspectives could well complement each other.

It has recently been shown that judgments about physical characteristics of social stimuli can be influenced by (implicit) attributions made by the observer about the agent’s intentions. For example, two studies reported that estimations of how far an agent’s head had rotated were influenced by the agent’s gaze direction [15], [16]. With gaze direction ahead of head rotation the head rotation was overestimated as compared to when the gaze was lagging behind head rotation. This perceptual bias seems to be induced by implicit attributions of the intention to either continue or discontinue to move in the direction of the head rotation. In a similar vein it was shown by Teufel and co-workers [17] that high-level mental attributions of ‘seeing’ versus ‘not seeing’ affect gaze adaptation differently. In this study, the agent faced left or right and wore highly reflective goggles. When observers believed the goggles were transparent, the gaze adaptation effect increased compared to when the observer believed the glasses were opaque. In both the Hudson et al. [15], [16] and Teufel et al. [17] studies, a mental state attribution affected low-level processing of the social stimulus. However, an important distinction is that in the Hudson et al. study [15] the attribution of the mental states occurred implicitly on the basis of the immediate perceptual history (participants did not recall having seen the gaze manipulation), whereas in the Teufel et al. [17] study the mental attributions reflected prior explicit knowledge. Both studies, however, emphasize that emotional/mental attributions affect very basic processing stages in social perception, and both highlight the bi-directional interaction between bottom-up and top-down information streams, which has been captured under the term ‘perceptual mentalizing’ [4]. Thus, even though mental/emotional attributions are generated by the perception of social stimuli [5], they in turn may influence the very perception of those social stimuli.

### Distortions in the Perception of Emotional Facial Expressions

We previously described a perceptual distortion of neutral facial expressions induced by the immediately preceding perceptual history of the face [18]. Neutral facial expressions were judged as slightly angry when they were immediately preceded by video-sequences in which the expression morphed from joy to neutral, while the identical neutral expressions were judged as slightly happy when the expression morphed from anger to neutral. Despite the neutral expressions being identical, they were perceived differently depending on the specific perceptual history. The participants were said to show a perceptual ‘overshoot’ bias. In the Jellema et al. study [18], these video-sequences were also used in an affective priming paradigm as task-irrelevant distractors, while the targets were formed by words with positive or negative valence superimposed on the last frame of the sequences. Participants had to make speeded evaluations of the word valence. The study found that positive words were identified faster as being positive when superimposed on the Anger-to-neutral videos, while negative words were identified faster as being negative when superimposed on the Joy-to-neutral videos. This indicated that the participants associated the Joy-to-neutral sequence with a negative valence and the Anger-to-neutral sequence with a positive valence. It further suggested that the perceptual ‘overshoot’ biases were not due to a cognitive response strategy, such as selecting the opposite of the start-emotion, but reflected a genuine shift in the observer’s perceptual judgment. The video-sequences were ecologically valid in that the velocities with which the facial expressions changed resembled those occurring during naturalistic dynamic facial expressions [19].

### The Contribution of Low-level Visual Mechanisms to Distortions in the Perception of Facial Expressions

It is still unknown what mechanism caused the overshoot response bias in the study by Jellema and co-workers [18]. In principle, one or more of a number of low-level visual mechanisms could, in a bottom-up stream, have contributed to the distorted perception of the neutral expression, including sequential contrast effects, representational momentum (RM), and adaptation.

In sequential contrast/context effects the target stimulus is influenced by the prior presentation of an inducing stimulus or anchor [20]. Thayer [21] showed that intensity ratings of both sad and happy facial expressions were enhanced when they were preceded by contrasting, as opposed to similar, facial expressions. Russell [22] presented two facial expressions of different identities side by side and found that the judgment of the first face (anchor) affected the judgment of the second face (target) in a repulsive manner. The results of this study were, however, criticized by Ekman and O’Sullivan [23], who maintained that the perception of emotion in facial expressions should be accurate and absolute, though they agreed that neutral or ambiguous expressions may be subject to contrast/context effects. Tanaka-Matsumi and co-workers [24] found that a neutral face (the target) was rated as less pleasant and less arousing when preceded by a happy face (the anchor) as compared to a sad anchor face. A common finding in these contrast/context effect studies was that it did not matter whether the identities of the actors depicting the emotion in the anchor and target stimuli were the same or different, the effect would always be there [21].

RM refers to the finding that the observer’s memory for the final position of a moving target is displaced further along the observed trajectory, and is hypothesized to reflect an anticipatory function [25], [26]. The extent of the memory displacement depends on the physical causes (e.g. gravity) and constraints (e.g. friction) that are inferred to act upon the object’s motion [27]. The displacement even occurs for static images of implied motion [28]. Although RM applies to the motion of physical objects, it can be influenced by psychological processes. For example, it can be influenced by conceptual knowledge the observer has about the nature of the object [29], and by the level of uncertainty about object behaviour [30]. RM might act on the gradual dynamic changes of the facial features, such as the U-shaped mouth in the happy expression, which morphs into a flat shape in the neutral expression and might then be extrapolated into an inverted U shape, giving the impression of a slightly angry face. However, it could, in principle, also act on an underlying positive-to-negative valence dimension. Yoshikawa and Sato [31] presented short video-clips depicting neutral expressions that morphed into intense emotional expressions, while observers had to judge the intensity of the expression depicted on the last frame, i.e. effectively the reverse of the videos used in the Jellema et al. study [18]. They found that the intensity of this final expression was overestimated and that the size of this effect was positively correlated with the speed of the intensity change. They explained the bias in terms of an RM effect, induced by the preceding sequence, but did not specify which dimension the RM effect was supposed to operate on.

Adaptation, which is ubiquitous in visual processing, is another candidate for explaining the overshoot response bias. A prolonged observation of a distorted face causes a normal (test) face to look somewhat distorted in the opposite direction. Face after-effects induced by adaptation have been reported for a variety of facial traits including emotional expressions [32], [33], and tend to generalise over different identities [34], [35]. Leopold and co-workers reported that periods shorter than 5 s failed to produce face after-effects [36], but many studies do not report whether durations shorter than 5 s were examined. Further, face aftereffects have been reported following just 500 ms adaptation [37]. Therefore, even though most adaptation durations in the literature are considerably longer than the presentation duration of emotional expressions in the study by Jellema and co-workers (470 ms) [18], adaptation cannot *a priori* be excluded as a candidate for explaining the overshoot effect.

### Emotional Anticipation

Another, more speculative, interpretation of the results of the study by Jellema and co-workers [18] is that the perceptual distortions were due to the observer keeping track of the immediate changes in the emotional state of mind of the actor. This led to an involuntarily anticipation of the most likely future emotional state, which might be referred to as ‘emotional anticipation’, i.e. the ability to involuntarily anticipate how the agent’s emotional state of mind will develop in the immediate future, based on the immediate perceptual history. It should be noted that we speak of ‘involuntary’ anticipation to indicate that the process is not initiated and/or guided by volition, but rather happens in a compulsory manner without involvement of volition, and independent of conscious control. It is not meant to reflect that the process happened *against* the participant’s volition, but simply in absence of volition. The anticipated emotional state would, in turn, affect (top-down) the evaluation of the target neutral expression. Thus, when an agent’s happy facial expression changes into a neutral expression the observer anticipates this change in emotional state to continue into a somewhat negative emotional state. Similarly, when an agent’s angry expression changes into a neutral expression the observer anticipates the agent’s emotional state of mind to become somewhat positive. This notion is corroborated by a recent fMRI study [38], which showed that happy-offsets and happy-onsets activate quite different brain areas, as do angry on- and offsets, even though the clips contained identical emotional expressions. These authors found that happy-offsets and angry-onsets activated areas related to threat and avoidance, while angry-offsets and happy-onsets activated areas related to reward and approach. The results of the affective priming experiment in the Jellema et al. study [18] also supported this notion as they indicated that happy-offsets were associated with a negative affective valence, and anger-offsets with a positive affective valence.

Emotional anticipation can be seen as a form of low-level mindreading [39], which is related to Theory of Mind (ToM), i.e. the ability to ‘read’ others’ mental states, such as beliefs, desires, intentions and emotions [40]. However, emotional anticipation does not necessarily rely on a ‘theory’, or high-level inferential processes, but might reflect an involuntary simulation process [13], in which the observer’s motor experience evoked by the perception of a dynamic facial expression facilitates the understanding of the expression.

The ability to instantaneously and involuntarily predict how the emotional state of mind of individuals may change across time is crucial for successful social interactions and also opens up possibilities for social manipulation [41]. It ties in with models that view perception as a ‘hierarchical predictive coding’ process [42], [43], which recently attracted renewed attention (see [44] for a critical discussion of hierarchical prediction models). Such models emphasize the importance of prediction for understanding both the animate and inanimate world surrounding us.

### The Current Study

Short video-clips were presented depicting intense emotional expressions (joy or anger) that morphed into a neutral expression, which then had to be evaluated. The aim of the current study was to investigate the mechanism(s) underpinning the perceptual distortion of the neutral expressions, by trying to experimentally disentangle the possible contributions of several bottom-up perceptual processes, and to examine whether top-down emotional anticipation processes might offer an alternative explanation. In the Jellema et al. study [18], no attempts had been made to elucidate the underlying mechanism. A series of experiments were designed specifically to address this issue. First, in Experiment 1 the endpoint of the clips was varied to test whether a crossing of the category-boundary would occur even when the endpoint belonged to the same category as the starting point, which will inform about the robustness of the perceptual distortion. In Experiment 2 the identity of the agent was changed at the end of the clips, which should not affect contrast/context effects, RM, or adaptation. However, the emotional anticipation explanation would predict a disappearance of the bias as the new identity is someone for whom no perceptual history is available. In Experiment 3 the sequences started from a neutral expression and morphed via happy (or angry) back to the same neutral expression. This condition further investigated a contribution of RM and contrast/context effects: the former would predict an overshoot, the latter the absence of a bias. Emotional anticipation predicts no bias, or possibly an undershoot (the emotion’s onset, directly followed by an offset, may inform about the actor’s emotional state of mind). Finally, in Experiment 4 a blank was inserted just before the final neutral expression to investigate whether disruption of the flow of motion present in the video-sequence would affect the evaluation due to a reset of low-level visual mechanisms.

## Experiment 1

It was previously reported that the perceptual judgments of neutral expressions depicted in the last frame of Joy-to-neutral and anger-to-neutral videos are characterized by an ‘overshoot’ bias [18]. In other words, the evaluations cross the category boundary. Experiment 1 aimed to investigate the strength and robustness of this perceptual distortion rather than the mechanism underpinning it. Hereto the endpoints of the videos were manipulated to so that they belonged to the same category as the starting points (both start and endpoint depicting joy, or both depicting anger). The rationale was that in order to obtain in this condition a perceptual distortion reflecting the ‘opposite’ emotion, it would have to involve a change in affective category (from joy to anger or vice versa), rather than a change from neutral to either joy or anger. Therefore, if in this condition the crossing of the category boundary would be found that would testify to the strength and robustness of this perceptual distortion.

### Method

#### Participants

Thirty-nine undergraduate Psychology students at Hull University (UK) took part in the experiment. All participants had normal or corrected-to-normal vision and received course credit for taking part. After applying exclusion criteria (see data reduction), the data of thirty-three participants were included in the analysis (age, M = 20.1 years, SD* = *3.2 years; 28 females).

#### Ethics statement

All participants taking part in one of the four experiments of this study provided their written consent. The study was approved by the Ethics committee of Hull University.

#### Stimuli

Pictures of eight actors displaying facial expressions of joy and anger were selected from the Pictures of Facial Affect (four males: EM, JJ, PE, WF, and four females: C, MO, PF, SW [45]). All faces were shown from frontal view with their eye gaze directed straight ahead. The photographs were in greyscale. The hair had been blackened so as to merge with the black background. The pictures were digitally adjusted to match in contrast and brightness. The eyes of all actors were positioned on approximately the same location on the screen. Faces measured about 13×20 cm when displayed on the screen, subtending roughly 8° vertically.

**Figure 1:**
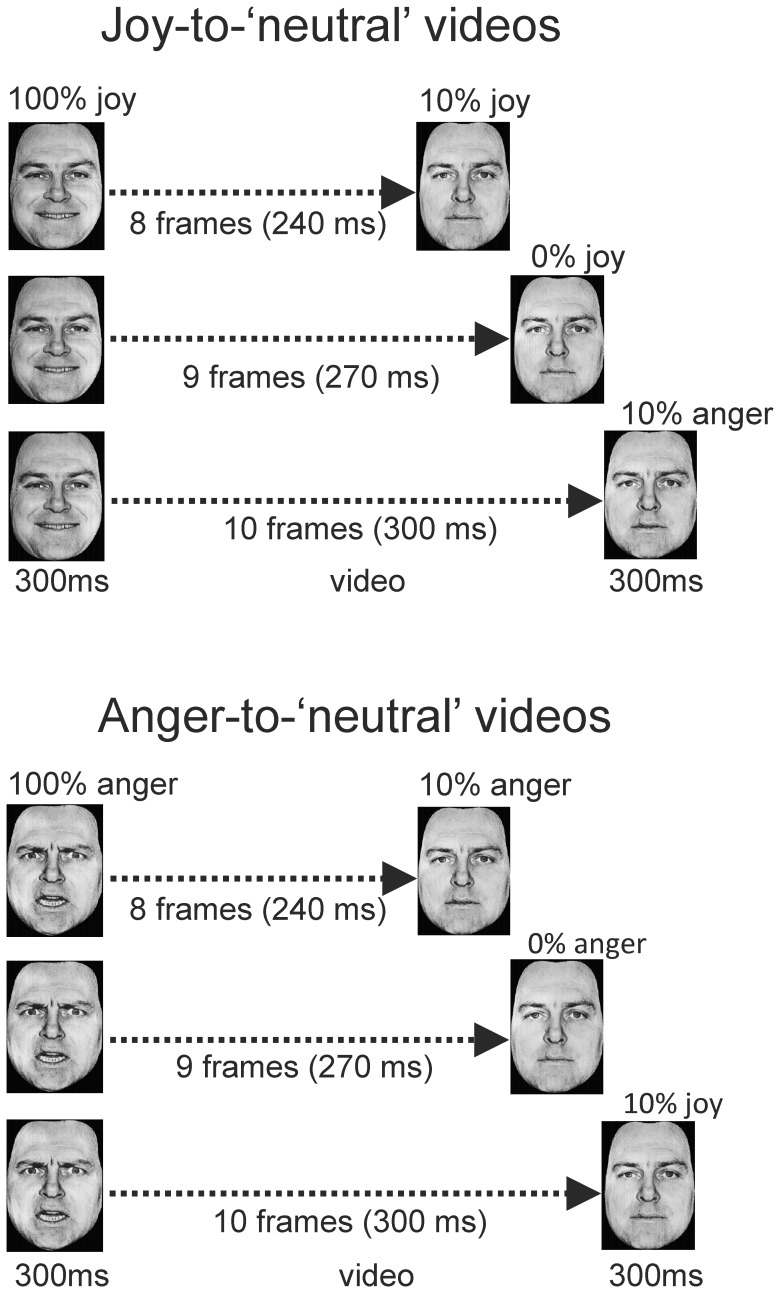
Illustration of the stimulus presentations in Experiment 1. Joy-to-‘neutral’ videos started with a facial expression of joy (100%), which gradually morphed into a 10% joy, a neutral or a 10% anger expression (top panel). Anger-to-‘neutral’ videos started with a facial expression of anger (100%), which gradually morphed into a 10% anger, a neutral or a 10% joy expression (bottom panel).

Nine interpolated images, in between the full-blown expression of joy or anger (which we call 100%) and the neutral expression (0%) were created at equal steps of 10% intensity change, using computer morphing procedures [46]. Rapid successive presentation of these interpolated frames constituted the videos. The first frame of each video sequence showed the emotional expression at 100% intensity and was presented for 300 ms to ensure the type of emotion was properly recognised [47]; the subsequent interpolated frames were shown for 30 ms each. The clips ended either in a neutral expression, in a 10% joy expression or in a 10% anger expression. The last frame remained on the screen for 300 ms. The total duration of the morph sequence was 270 ms (9×30 ms), and was 30 ms longer or shorter for clips with 10% intensity endpoints (see Figure 1). A total of 48 videos were made (8 actors×2 perceptual histories×3 endpoints).

Expressions of joy and anger were selected, rather than joy and sadness, on the basis of the following considerations. First of all, in terms of the agent expressing the emotion, joy and anger are both approach-oriented emotions, whereas sadness - and also fear - are avoidance-oriented emotions. However, in terms of the observer, joy is approach-oriented while the negative emotions anger, sadness and fear are avoidance-oriented [48], [49]. Ideally, the start emotions should be equal (1) in terms of arousal and (2) in terms of the extent to which their distinctive physical features differ geometrically from the neutral expression. This is important as any of these two factors could in principle contribute to differences in response biases when comparing different emotions. According to the Circumplex model of affect [22], in which emotions are represented by values on the continuous dimensions of arousal and pleasantness, joy and anger are more similar in terms of arousal than joy and sadness, while they differ maximally in pleasantness. The extent of geometrical change in the face when morphing from 100% intensity to neutral should be identical for the different emotional expressions used, to exclude the possibility that an unequal extent of extrapolation of facial curves resulted in different response biases. Joy and anger are in this respect fairly similar, whereas happy and sad are not (the sad expression is considerably less expressive than the happy and angry ones [50]). The above considerations render the sad expression unsuitable for use on the 5-point scale opposite to joy. The considerations about the extent of geometrical change in the face would also exclude the expression of fear as start emotion. The expression of surprise was considered inappropriate because of its twofold emotional valence [51], while disgust is less recognizable, as it may present a mixture of emotion.

#### Measurement of the perceptual evaluations: the response scale

The participants’ perceptual judgments of the facial expressions were measured on a scale ranging from slightly angry (1) via neutral (3) to slightly happy (5), where written labels indicated the different facial expressions and their intensities [18]. Responses were selected by pressing one of the 5 labeled keys on a button box (SR-Box, Psychology Software Tools, Inc., USA). In principle, a scale consisting of different pictures of facial expressions from which participants had to select the expression that most resembled the expression at the end of the clip could be used [30]. However, given the current experimental purposes, such a scale would be problematic. The process of going through the sequence of pictures showing different intensities of the emotional expressions of joy and anger, e.g. by using an interactive slider [30], would, unwillingly, create a new perceptual history, which would interfere with the experimental perceptual history. A procedure in which participants select, out of two different test images of facial expressions, the expression that best resembled the clip’s end-expression would also not be appropriate, as these test images could induce contrast effects themselves.

It should be noted that the response scale was not a forced two-choice scale as participants could select the middle point of the scale, corresponding to a neutral expression. Further, replacing the labels ‘slightly angry’ and ‘slightly happy’ by the labels ‘slightly negative’ and ‘slightly positive’ produced a very similar overshoot response bias, as was shown in a separate experiment (7 females, 1 male; age, M = 20.4 years, SD* = *2.5 years; Joy-to-neutral: M = 2.65, SD = 0.33, Anger-to-neutral: M = 3.46, SD = 0.54; t(7) = −4.86, p = .002).

#### Experimental procedure

Participants were seated at a viewing distance of 80 cm from a monitor (17-inch, 1024×268 pixels, 100 Hz). The stimuli were presented using E-Prime (v. 1.2; Psychology Software Tools, Inc.). First a calibration condition was presented, in which the eight actors were shown with static neutral expressions, i.e. neutral expressions according to the ratings from Ekman and Friesen’s study [45]. Each calibration trial started with a fixation cross displayed in the centre of the screen for 500 ms, followed by a single frame depicting the neutral face, presented for 600 ms. Sixteen calibration trials were presented (8 actors, 2 repetitions each) in random order. Participants were prompted to rate these ‘neutral’ expressions using the 5-point scale.

After the calibration trials, 8 practice trials were completed, followed by 64 randomised experimental trials, half of which started with a happy expression (called joy-to-‘neutral’ videos) and half with an angry expression (called anger-to-‘neutral’ videos). Of both types of videos, 50% of trials ended with the neutral expression, 25% of trials ended with the 10% joy and 25% of trials with the 10% anger expression. Each trial started with the fixation cross (500 ms), followed by the video. As soon as the video ended a blank screen appeared with a prompt to provide a response. The participant’s task was to evaluate the last expression using the same 5-point scale as for the calibration trials. Participants were instructed that their RTs were irrelevant, but that they should give their response within 3 seconds. The duration of the entire experiment was 20 minutes.

#### Data reduction and analysis

Trials in which the RTs fell below 250 ms or above 3000 ms were considered outliers and were removed (4.8%). Participants were excluded if more than 25% of the RTs values were outside this range (n = 4). We further applied a ±2.5 SD rule to the mean difference ratings (i.e. mean rating in Anger-to-neutral condition minus mean rating in Joy-to-neutral condition), which excluded 2 more participants. The calibration scores were used to adjust the scores obtained in the experimental trials; a calibration factor [3.00 minus the calibration score] was added to the raw scores. This allowed performing one-sample t-tests with test value 3.00 in the two perceptual histories. All statistical analyses were performed on the calibrated scores.

### Results

**Figure 2:**
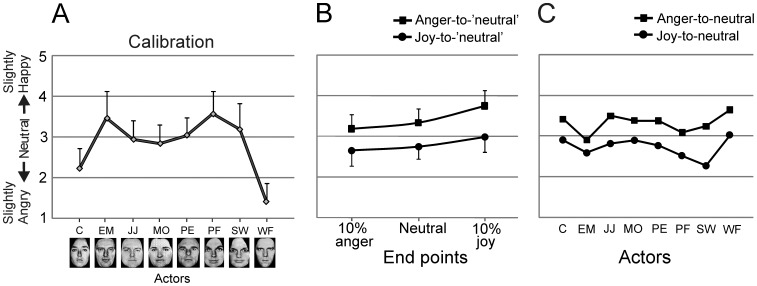
Results of Experiment 1. (A) Ratings on the 5-point scale (y-axis) for the neutral expressions of the eight actors (x-axis) in the calibration phase. Error bars indicate +1SD. (B) Ratings on the same scale for the expressions depicted on the last frame of the joy-to-‘neutral’ and anger-to-‘neutral’ videos. The sequences ended at 10% anger, neutral or 10% joy (x-axis). Error bars indicate 1SD. (C) Ratings for exclusively the neutral expressions at the end of the joy- and anger-videos for each of the eight actors to illustrate response consistency across actors.

The mean calibration scores for the neutral expression of each actor are shown in Figure 2A. A very similar pattern of calibration scores was obtained across experiments, with the neutral expression of actors C and WF systematically rated as slightly angry. The main results are shown in Figure 2B and C. A 2×3 repeated measures ANOVA with Perceptual history (joy-to-‘neutral’ video vs. anger-to-‘neutral’ video) and Endpoint (10% joy vs. neutral vs. 10% anger) as within-subject factors, showed a robust significant main effect for Perceptual history (F(1,32)_ = _67.39, p<.0001, η_p_^2^ = .678). The expression in the last frame was perceived as more angry in the joy videos (M = 2.81, SD = 0.34) as compared to the anger videos (M = 3.43, SD = 0.35). There was also a significant main effect for the factor Endpoint (F(2,64)_ = _58.98, p<.0001, η_p_^2^ = .648), with expressions in the 10% anger endpoint condition evaluated more negatively than those in the neutral endpoint condition (t(32) = −3.32, p = .002), and expressions in the neutral endpoint condition more negatively than those in the 10% joy endpoint condition (t(32) = −8.05, p<.001). The Endpoint by Perceptual history interaction was significant (F(2,64)_ = _3.77, p = .028, η_p_^2^ = .105). In the neutral endpoint condition, the mean ratings in the joy videos (M = 2.76, SD = .30) and anger videos (M = 3.34, SD = .33) differed significantly from each other (t(32) = −6.45, p<.0001), and each of them differed significantly from 3.00 (joy-video: t(32) = −4.67, p<.001; anger-video: t(32) = 5.94, p<.001; two-tailed). Remarkably, the 10% joy endpoint in the joy-videos was rated as significantly more angry than the 10% anger endpoint in the anger-videos (t(32) = 2.34, p = .025), despite the former expression being 20% more ‘positive’ than the latter.

### Discussion

In Experiment 1 videos starting with an expression of joy or anger ended either with a neutral, a 10% joy or a 10% anger expression. The results showed a robust overshoot response bias: the neutral expression in the last frame of the joy-to-‘neutral’ videos was judged as slightly angry, while the identical neutral expression in the last frame of the Anger-to-neutral videos was judged as slightly happy, confirming the results by Jellema et al. [18]. This finding was quite consistent across actors. Importantly, the conditions in which the endpoint was not neutral, but 10% joy or 10% anger, demonstrated that the response bias was strong enough to overcome a 20% difference in emotional intensity, as the 10% joy endpoint in the joy-videos was rated as significantly more angry than the 10% anger endpoint in the anger-videos. In the following three experiments the mechanism underpinning this perceptual distortion of facial expressions induced by the perceptual history was investigated.

## Experiment 2

In Experiment 2 the identity of the actor was changed in the last frame of the video-clips. Therefore the judgment of the final expression was made from an identity that was new to the observer; i.e. someone for whom no immediate perceptual history was available. The rationale was that if the overshoot bias resulted from sequential contrast/context effects then a change in identity should not affect the bias, as the contrast remained intact. Contrast effects for emotional facial expressions have been shown to be immune to a change in identity between the anchor and target stimuli (e.g. [24]). Similarly, if the overshoot bias would result from RM on an underlying positive-negative valence dimension, then a change in identity should not affect it, as the underlying valence dimension remains intact. If the bias was due to adaptation to the intense facial expression in the first frame (300 ms), resulting in an aftereffect in opposite direction, then a change in identity should again not affect the bias, as facial expression aftereffects generalise over different identities [34], [35]. Thus, if the identity change would remove, or largely reduce, the response bias, than that would suggest that none of these low-level visual mechanisms played a major role in generating the bias. Bias removal would, however, be consistent with an emotional anticipation account. Emotional anticipation would predict the absence of an overshoot bias as the observer cannot anticipate the emotional state of someone on the basis of a perceptual history that pertains to a different identity.

Even though care was taken to limit the perceptual shift due to the identity transition, a certain amount of shift was unavoidable. In theory, this might have disrupted the perceptual flow causing the perceptual system to ‘reset’ [52]. Therefore, in addition, a version of Experiment 2 was conducted in which the identity change was smoothed.

### Method

#### Participants

The two (instant and smooth) versions of Experiment 2 were performed on different groups of undergraduate Psychology students at Hull University. After applying exclusion criteria (see below), 23 participants were included in the instant-identity-change experiment (age, M = 19.3 years, SD* = *1.53 years; 19 females) and 20 participants in the smooth-identity-change experiment (age, M = 19.4 years, SD* = *2.56 years, 17 females). None of the participants took part in any of the other experiments. All had normal or corrected-to-normal vision, gave informed consent, and received course credit for taking part.

#### Stimuli

**Figure 3:**
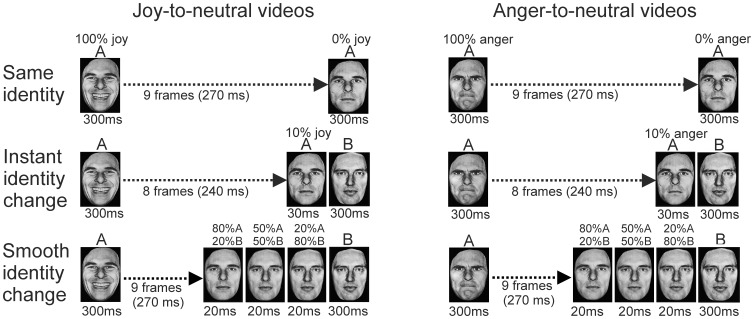
Illustration of the stimulus presentations in Experiment 2. Examples of the same-identity condition (top), the instant identity-change condition (middle) and the smooth identity-change condition (bottom), are given for joy-to-neutral (left side) and anger-to-neutral (right side) perceptual histories.

In the instant identity-change experiment, videos started with an actor (identity-A) displaying a 100% emotional expression (joy or anger), which morphed into a 10% expression, after which the identity changed instantaneously into identity-B (0%, neutral expression; Figure 3). Thus the identity changed from the last but one to the last frame. The entire duration of the clip was 870 ms (first frame for 300 ms, 9 interpolated frames for 30 ms each, and last frame for 300 ms; Figure 3, middle panel). The face images of identities A and B were resized to match in outer dimensions as much as possible, with the eyes presented at a fixed location on the screen, to limit the extent of geometrical shift between identities.

In the smooth-identity-change experiment, the identity transition was morphed by creating three interpolations between identity-A at 10% happy or angry and identity-B at 0%, and inserting these in the sequence directly before the last, neutral, frame (Figure 3, bottom panel). The three inserted frames consisted of 80%A-20%B, 50%A-50%B and 20%A-80%B. Each lasted 20 ms, increasing the total duration of the sequence to 930 ms Each actor was combined with four other actors, two of which were of the same and two of opposite sex.

In the control condition the same identity was shown throughout the clip (same-identity condition), from 100% joy or anger to neutral, as in Experiment 1 (Figure 3, top).

#### Experimental procedure

The experiments started with a calibration phase, in which the participants rated the static neutral expression of each actor presented for 600 ms, using the same 5-point scale as in Experiment 1. Following eight practice trials, 64 experimental (identity-change) and 64 control (same identity) trials were presented in random order (8 actors×2 perceptual histories×4 repetitions). Further procedures and apparatus were as in Experiment 1.

#### Data reduction

Exclusion criteria were as in Experiment 1. Trials in which the RTs fell below 250 ms or above 3000 ms were removed (4.9% in the instant-change and 6.9% in the smooth-change experiment). Participants were excluded if more than 25% of the RTs values were outside this range (n = 2, both experiments). The ±2.5 SD rule applied to the mean difference ratings excluded another three participants (one in the instant-change and two in the smooth-change experiment).

### Results

#### Instant identity-change experiment

A 2×2 ANOVA with Perceptual history (joy-to-neutral vs. anger-to-neutral) and Identity change (no change vs. change) as the within-subject factors showed significant main effects of Perceptual history (F(1,22)_ = _41.2, p<.001, η_p_^2^ = .652) and Identity change (F(1,22)_ = _7.9, p = .01, η_p_^2^ = .264). Importantly, the interaction factor was highly significant (F(1,22)_ = _18.8, p<.001, η_p_^2^ = .461). Similar to Experiment 1, in the same-identity condition, neutral faces were perceived as more angry in the joy videos (M = 2.82, SD = 0.23) than in the anger videos (M = 3.32, SD = .26), (t(22) = −7.13, p<.001), and each differed significantly from 3.00 (joy: t(22) = −3.69, p<.001; anger: t(22) = 5.87, p<.001). However, in the identity-change condition the ratings for the neutral expressions did not differ between the two perceptual histories (t(22) = −1.15, p = .263). Thus, a change of identity of the actor in the last frame of the video-sequence effectively removed the overshoot bias.

#### Smooth change in identity

**Figure 4:**
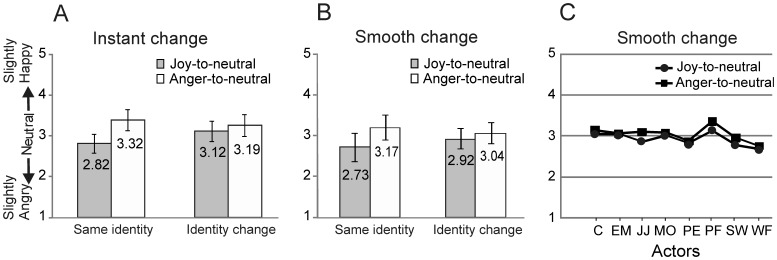
Results of Experiment 2. (A) Instant identity-change. Scores for the neutral expressions at the end of the joy-to-neutral and anger-to-neutral sequences in the same-identity and instant identity-change conditions. (B) Smooth identity-change. Same as in (A) but for the smooth identity-change condition. Error bars indicate ±1SD. (C) Ratings for each of the eight actors in the smooth identity-change condition to illustrate the response consistency across actors.

A 2×2 ANOVA, similar to the one performed for the Instant identity-change experiment, showed a main effect of Perceptual history (F(1,19)_ = _75.3, p<.001, η_p_^2^ = .78) and no significant main effect of Identity change (F(1,19)_ = _.25, p = .62, η_p_^2^ = .013). The interaction factor was highly significant (F(1,19)_ = _33.9, p<.001, η_p_^2^ = .46). In the same-identity condition the perceptual histories again differed significantly from each other (t(20) = −8.68, p<.001) and from 3.00 (p’s <.01). In the smooth identity-change condition, even though the mean ratings for the neutral expression in the joy and anger-videos overlapped considerably (Figure 4C), the ratings in two perceptual histories did differ significantly from each other t(19) = −4.21, p<.001). However, neither perceptual history differed from 3.00 (Joy, t(19) = −1.5, p = .15; Anger, t(19) = .72, p = .48).

### Discussion

Experiment 2 showed that for an overshoot response bias to occur, the identity of the actor should remain the same during the perceptual history: a change in identity in the last frame of the sequence effectively removed the bias. The removal of the bias was absolute when the identity change was instantaneous, while some bias still seeped through when the identity-change was smoothed. The most likely explanation for the latter is that the participants in some cases did not notice the change in identity due to the morphing procedure and believed actors A and B to be one and the same person. In contrast, in the instant condition the identity change was always noticed. This was supported by debriefing of participants. Debriefing was done for 8 of the 20 participants following the smooth identity change condition. All 8 participants indicated that they believed a change in identity had occurred in some, but not in all, trials.

The absence of an overshoot bias in the identity-change condition suggests that the large bias in the same-identity condition was not caused by sequential contrast effects, as the contrasts were not affected by the identity change. It also suggests that RM on an underlying positive-negative valence dimension did not cause the overshoot bias, as an identity change does not change the underlying valence dimension.

The results seem further to rule out adaptation as a major cause of the bias as the change in identity should not dramatically affect the aftereffects induced by adaption to a facial expression. Although some decrease in aftereffect due to identity change is reported [34], [35], a significant adaptation effect should still be present. However, the instant identity-change experiment revealed no overshoot bias whatsoever. It is important to rule out adaptation because face aftereffects following very short adaptation durations (500 ms) have been reported, especially at higher, position-invariant, levels of the visual system [37]. Moreover, many studies reporting aftereffects following adaptation of 5 s or more did not indicate whether periods shorter than 5 s were ineffective. The lack of a contribution by adaptation is further corroborated by the previous finding that a three-fold increase in the duration of the first frame did not affect the extent of overshoot bias [18].

The results are compatible though with top-down emotional anticipation, involving integration of emotion and identity information, which was available in the immediate perceptual history. Actor B in the last frame was someone for whom no perceptual history had been witnessed, so the observer did not know anything about B’s emotional state other than that B had a neutral expression, and therefore rated B as neutral.

## Experiment 3

Experiment 3 further assessed a possible contribution of contrast effects to the overshoot response bias using a different rationale. The video-sequence was modified so that it started from a neutral expression and morphed via an expression of maximal joy or anger back to the same neutral expression (called Loop condition). If the response bias found in the previous experiments was due to the contrast between the to-be-evaluated expression in the last frame and the ‘anchor’ stimulus in the first frame (both presented for 300 ms), then in the loop condition one would expect the last neutral expression to be judged as neutral and not to obtain an overshoot bias, even though the second half of the sequence (joy/anger-to-neutral) is identical to the sequence in the previous experiments.

Furthermore, RM would predict an overshoot (continuation of the implied motion), while emotional anticipation would predict no bias, or possibly even an undershoot response.

### Method

#### Participants

Twenty-eight undergraduate Psychology students at Hull University took part in the experiment. After applying exclusion criteria (see below), twenty-four participants were included in the analysis (age, M = 19.9 years, SD* = *2.04; 15 females). None of the participants took part in any of the other experiments. All had normal or corrected-to-normal vision, gave informed consent, and received course credit for taking part.

#### Stimuli

**Figure 5:**
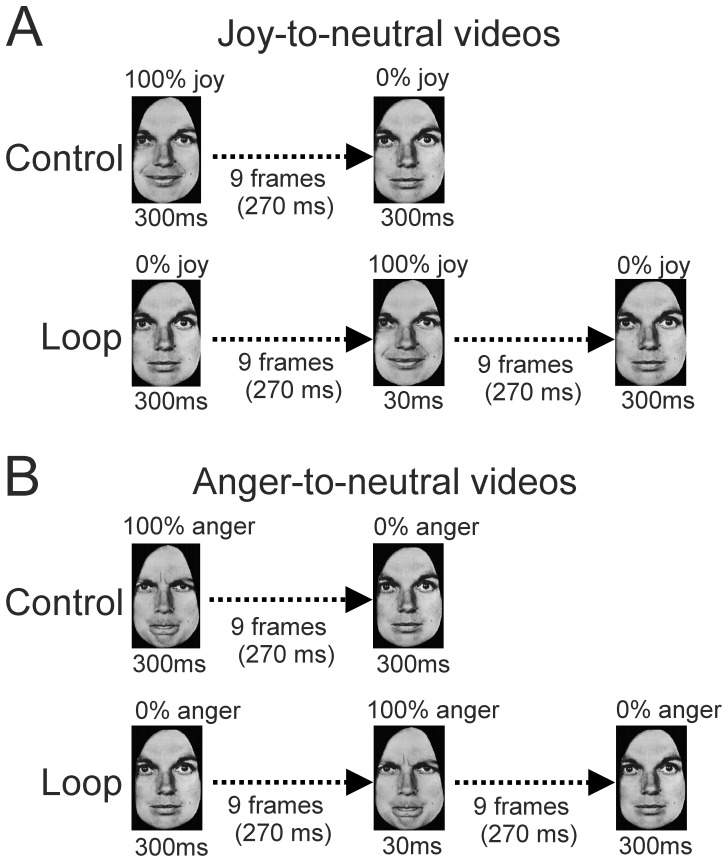
Illustration of stimulus presentations in Experiment 3. (A) Joy-to-neutral sequence in the control condition (top row) and neutral-to-joy-to-neutral sequence in the loop condition (bottom row). (B) Similar sequences but for anger-to-neutral (top row) and neutral-to-anger-to-neutral (bottom row) conditions.

Video-clips of eight actors, which started with a neutral expression, and morphed via an expression of intense joy or anger back to the same neutral expression, were created. The morphing sequence consisted of nineteen interpolated frames, each 30 ms long (Figure 5). The first and the last frames both lasted 300 ms, making the entire sequence last for 1170 ms. The stimuli used for the control condition were the same as those used in the same-identity condition in Experiment 2.

#### Experimental procedure

Participants first rated the static neutral expression of each actor presented for 600 ms using the 5-point scale. Following eight practice trials, one experimental block was presented with 96 randomized trials (8 actors×2 perceptual histories×2 conditions×3 repetitions). The participant’s task was to evaluate the last frame of the video-clips on the same 5-point scale. Further procedures and apparatus were as in Experiment 1.

#### Data reduction

Exclusion criteria were as in Experiment 1. Trials in which RTs fell below 250 ms or above 3000 ms were removed (7.9%). Participants were excluded if more than 25% of the RTs values fell outside the mentioned range (n = 3) and when the mean difference rating fell outside a ±2.5 SD range (n = 0).

### Results

**Figure 6:**
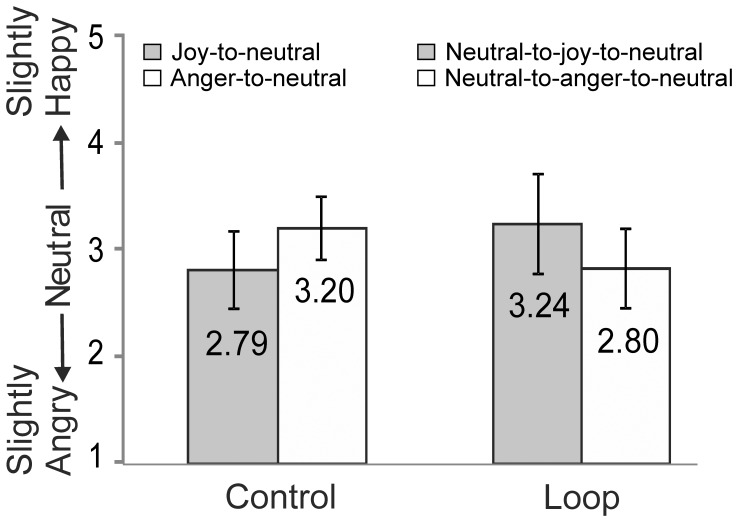
Results of Experiment 3. Ratings of the neutral expressions at the end of the video-sequences in the Control and Loop conditions. Error bars indicate ±1SD.

The 2×2 ANOVA with Condition (No loop vs. loop) and Perceptual history (Joy-to-neutral vs. Anger-to-neutral) as the within-subjects factors showed no significant main effects for Condition (F(1,23)_ = _.35, p = .56, η_p_^2^ = .015) and Perceptual history (F(1,23)_ = _.24, p = .88, η_p_^2^ = .001), but a highly significant Condition by Perceptual history interaction (F(1,23)_ = _21.4, p<.0001, η_p_^2^ = .48; Figure 6). In the control (No loop) condition, the typical overshoot effect was found; the mean ratings of the neutral faces was 2.79 in the Joy-to-neutral and 3.20 in the Anger-to-neutral sequences, which differed significantly from each other (t(23) = 4.18, p<.0001). In the Loop condition the reversed pattern emerged: the neutral expression at the end of the Neutral-to-joy-to-neutral video was evaluated as slightly happy (M = 3.24, SD = 0.53), and at the end of the Neutral-to-anger-to-neutral videos as slightly angry (M = 2.80, SD = 0.47). These evaluations differed significantly from each other (t(23) = 2.60, p = .016) and each differed significantly from 3.00 (Neutral-joy-neutral: (t(23) = 2.50, p = .020); Neutral-anger-neutral (t(23) = −2.16, p<.041; two tailed). The reversal of the response bias (“undershoot” bias) was consistently found across actors.

### Discussion

Experiment 3 established that the contrast between the initial frame (300 ms duration) and the last, to-be-evaluated, frame (300 ms) did not play a significant role in bringing about the overshoot response bias. In the Loop condition there was no contrast between the initial and last frames (both showed identical neutral expressions) and therefore the sequential contrast hypothesis predicted the absence of a response bias. However, a response bias was obtained, but one that was opposite in direction (undershoot bias) to the one in the control condition.

The finding that the joy/anger-to-neutral videos produced an overshoot response, while the Loop videos produced an undershoot response, is also not compatible with RM as underpinning mechanism. RM would predict an overshoot bias as RM involves a continuation of the immediately preceding direction of motion, attributed to the object’s implied momentum [26], [27]. It seems unlikely that RM might have induced the expectation that another, identical, loop would occur, as the implied momentum is determined by the direction of motion immediately preceding the stopping point. Similarly, RM would predict the joy/anger-to-neutral videos to extrapolate into an ‘overshoot’ rather than into a repetition of the same joy/anger-to-neutral sequence.

The reversed response bias is compatible with emotional anticipation. It is possible that observers perceived the brief smile in the joy loop as a positive social sign, reflecting the agent’s positive attitude towards them, and consequently evaluated the agent positively, as we tend to like people who like us [53]. Similarly, the brief frown in the anger loop may have constituted a negative social sign resulting in the notion that the agent held a negative disposition toward the observer, and hence the negative evaluation of the neutral expression. Crucially, the emotional anticipation interpretation takes into account the agent’s perceptual history, which is the content of the entire video-sequence, not just the second half of it (which part was identical to the control condition). The perceptual histories differed markedly between the Loop and control conditions and therefore different outcomes were anticipated. In the Loop videos the emotion onset was immediately followed by an offset, whereas in the control videos only the emotion offset was shown. The offset of joy, even though it contains the expression of joy, does not display a positive social signal, but rather a negative one, as joy offsets are threat related [39].

## Experiment 4

In the video-sequences presented so far, many geometrical features in the face changed in a continuous and smooth manner. To examine to what extent the overshoot response depended on the continuous, uninterrupted flow of motion present in the video-sequence up to the to-be evaluated neutral frame, in Experiment 4 the video-sequence was disrupted by inserting a mask (400 ms duration) immediately before the last, neutral, frame. Disruption of the flow of motion might reset low-level visual mechanisms [52]. If the overshoot bias depended on such continuous motion then this manipulation should remove the bias. However, insertion of the mask would not affect the anticipation of the emotional state of the actor, and therefore the emotional anticipation explanation would predict to find an overshoot bias.

In addition, Experiment 4 acted as a control for the Instant identity-change condition in Experiment 2, in the sense that it allowed to examine whether the relatively abrupt change in low-level features caused by the instant identity change might, in itself, have caused the removal of the overshoot bias (possibly through a reset of low-level visual mechanisms).

### Method

#### Participants

Sixteen undergraduate Psychology students at Hull University took part. After applying exclusion criteria, fifteen participants were included in the analysis (age, M = 20.2 years, SD* = *3.0; 12 females). None of the participants took part in any of the other experiments. All had normal or corrected-to-normal vision, gave informed consent, and received course credit for taking part.

#### Stimuli

**Figure 7:**
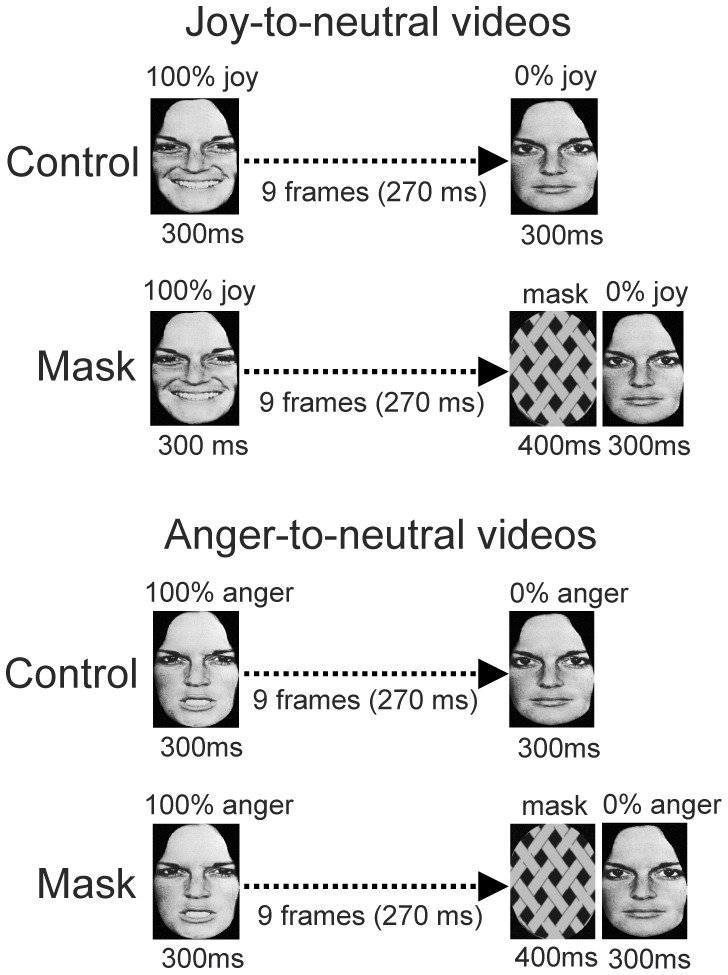
Illustration of the stimulus presentations in Experiment 4. Joy-to-neutral videos (top panel) and Anger-to-neutral videos (bottom panel) in the Control and Mask conditions are shown.

In the Mask condition, a 400 ms long mask, consisting of an oval grid in grey scale, was inserted in the Joy-to-neutral and Anger-to-neutral video-clips directly before the last frame (Figure 7). The contour of the oval shape matched that of the faces. Due to insertion of the mask the total duration of the sequence was 1270 ms; all other stimulus parameters were as in Experiment 2. The control condition was identical to the mask condition except that no mask was inserted (Figure 7).

#### Experimental procedure

The procedure and apparatus were the same as described for the previous experiments except that the experimental block consisted of 64 trials, 32 for the control and 32 for the mask condition (8 actors×2 perceptual histories×2 repetitions), presented in random order.

#### Data reduction

Exclusion criteria were as in previous experiments. Trials in which the RTs fell below 250 ms or above 3000 ms were removed (8%). Participants were excluded if more than 25% of the RTs values were out of the mentioned range (n = 0). The ±2.5 SD rule applied to the mean difference ratings excluded one participant.

### Results

**Figure 8:**
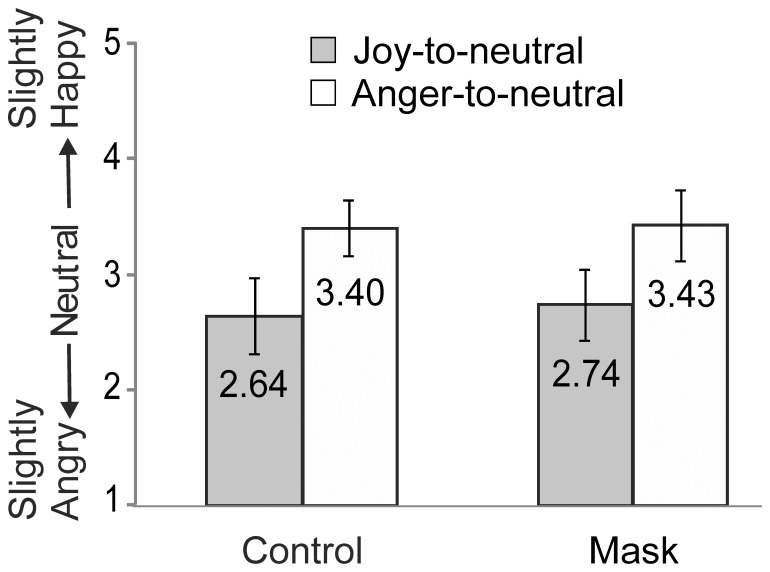
Results of Experiment 4. Mean scores for the neutral expressions at the end of the Joy-to-neutral and Anger-to-neutral video-clips without an inserted mask (control) and with (mask). Error bars indicate ±1SD.

A 2×2 ANOVA with Condition (control vs. Mask) and Perceptual history (Joy-to-neutral vs. Anger-to-neutral) as factors showed a robustly significant main effect of Perceptual history (F(1,14)_ = _47.50, p<.001, η_p_^2^ = .77). The factor Condition was not significant (F(1,14)_ = _1.86, p = .194, η_p_^2^ = .117), and the Condition by Perceptual history interaction (F(1,14)_ = _1.68, p = .215, η_p_^2^ = .107) was not significant (Figure 8). In the control (No mask) condition, the ratings for the neutral expression in the two perceptual histories differed significantly from each other (t(14) = −6.76, p<.0001), and each differed significantly from 3.00 (joy: t(14) = −4.24, p<.001; anger: t(14) = 6.57, p<.001). Similarly, in the Mask condition the two perceptual histories differed significantly from each other (t(14) = −6.55, p<.0001), while each differed significantly from 3.00 (joy: t(14) = −3.28, p<.005; anger: t(14) = 5.42, p<.0001).

### Discussion

Experiment 4 showed that the overshoot bias survived the insertion of a 400 ms long mask. The extent of bias in the conditions with and without mask was very similar. As this manipulation will have reset low-level perceptual mechanisms that rely on the continuous flow of motion [52], we conclude that such mechanisms did not play a crucial role in bringing about the overshoot bias. The results further indicate that the absence of an overshoot bias in the Instant identity-change condition in Experiment 2 was not related to the relatively abrupt transition between the two identities, as the insertion of the mask introduced a considerably more abrupt change in low-level parameters than the instant identity-change did.

## General Discussion

In the current study perceptual judgments were made about facial expressions, the perceptual histories of which involved a gradual dynamic transition from one expression into another for one and the same identity. This made the stimuli more ecologically-valid than those in studies of contrast/context effects, in which the perceptual history typically consists of a static anchor stimulus, while the identities of anchor and target face differ [21], [24]. The current approach therefore opened up the possibility for ‘emotional anticipation’ (also referred to as ‘low-level mindreading’ [39]) to influence top-down the perception of the target stimulus. In four experiments we examined whether the distortions could be explained by low-level visual mechanisms, and whether emotional anticipation processes could have contributed.

In Experiment 1, the immediate, dynamic perceptual history biased the perception of the neutral expression depicted at the end of the video-sequence. Identical neutral facial expressions were consistently judged as more angry when presented at the end of Joy-to-neutral videos than when presented at the end of Anger-to-neutral videos. The distortion was strong enough to overcome a 20% difference in emotional intensity, as the 10% joy endpoint in the joy-videos was rated as significantly more angry than the 10% anger endpoint in the anger-videos.

One interpretation of these results is that observers involuntarily kept track of the emotional state of mind of the actor and implicitly anticipated what the actor’s emotional state would be in the immediate future. Such a top-down influence could bias the visual perception of the current facial expression in the direction of the anticipated emotional state of mind, i.e. the ‘emotional anticipation’ hypothesis. However, a number of basic perceptual mechanisms, such as sequential contrast/context effects, representational Momentum (RM), and adaptation, might in principle also be able to explain the bias in perceptual report. Therefore, in Experiments 2 to 4 the viability of these low-level explanations was tested.

In Experiment 2 a change in facial identity just before the final neutral expression was reached effectively removed the overshoot bias. This suggested that sequential contrast/context effects probably did not contribute to the bias, as the degree of contrast between the first and last frames of the videos remained unaffected by the identity change. It also argued against an explanation in terms of RM on an underlying positive- negative valence dimension, as the underlying valence dimension was not affected by the manipulation.

Experiment 3 was designed to further assess whether the initial, emotionally intense expression (presented for 300 ms), might have given rise to a sequential contrast effect with the final neutral expression (also presented for 300 ms). As the ‘loop’ sequence in this experiment started with a neutral expression (300 ms), according to the sequential contrast hypothesis the final neutral expression should be perceived as neutral. However, the last neutral frame was judged as slightly happy after the ‘joy loop’ and as slightly angry after the ‘anger loop’ (undershoot response bias), which argued against the sequential contrast explanation. It also argued against RM operating on a positive-negative valence dimension and RM operating on facial features, as the latter explanations predicted an overshoot bias.

In Experiment 4 the overshoot bias was found despite the insertion of a 400 ms long mask immediately before the last, neutral expression. This indicated that the overshoot bias did not depend on a low-level visual mechanism generated by the continuous flow of motion in the video-sequence, as such a mechanism would have been ‘reset’ by the mask insertion [52].

Taken together, these manipulations suggest that the overshoot bias found in Experiment 1 was not predominantly caused by bottom-up perceptual mechanisms. A sequential contrast/context effect [20], [21], [24] is an unlikely explanation as a change in identity removed the overshoot (Experiment 2), while a neutral starting-point did not result in a neutral evaluation of the endpoint (Experiment 3). Adaptation can be ruled out as the change in identity should not considerably affect the adaptation-aftereffect [34], while this manipulation effectively removed the overshoot (Experiment 2). RM operating on an underlying positive-negative valence dimension is an unlikely explanation as it predicts that a change in identity would not affect the bias yet it did (Experiment 2). Further, it would predict an overshoot bias in the ‘Loop’ experiment, whereas the opposite, i.e. an undershoot bias, was elicited (Experiment 3). RM on the basis of changing geometrical facial features was rendered unlikely by the finding of a removal of the overshoot bias after a change in identity (Experiment 2). This latter explanation can be further discredited by considering the extent of geometric change in the two perceptual histories. Happy facial expressions typically present a U-shaped mouth with pulled-up lip corners [54]. Thus, in the happy-to-neutral morphs, the change of the U-shaped mouth into the flat mouth (neutral expression) might have been extrapolated in the observer’s mind into a slightly inverted U-shaped mouth (denoting slight anger). However, the shape of the mouth in the angry expressions was often flat, as lips were pressed together. Nevertheless, in the Anger-to-neutral condition an equally large overshoot bias was found as in the Joy-to-neutral condition. Changes in the height of the eye brows, which are typically lowered in angry expressions, can also not explain the response bias as in Joy-to-neutral videos the eyebrow height hardly changed, yet a large overshoot bias was induced.

The above low-level visual mechanisms do not seem able to satisfactorily explain the overshoot response bias. The findings are, however, compatible with an emotional anticipation mechanism. That is, the observer, involuntarily, keeps track of any changes in the agent’s emotional state of mind (based on the agent’s facial expressions and identity information) and continually updates its prediction, or anticipation, of what the most likely next emotional state of mind of the agent might be.

### Possible Mechanisms Underpinning Emotional Anticipation

Widely different models have been proposed for the way in which we understand and anticipate others’ emotional/mental states. In philosophy of mind, the two main approaches to intersubjective understanding are often referred to as theory theory (TT) and simulation theory (ST). According to TT the attribution of emotional/mental states reflects the theoretical use of folk psychology, psychological laws and tacit knowledge [55]. ST might involve either an explicit simulation (i.e. deliberately putting oneself in the other’s shoes [56], or an implicit (subpersonal) ‘embodied simulation’, i.e. simulating others’ actions and emotions in one’s own sensorimotor system [57]. ST received a huge boost by the discovery of mirror neuron mechanisms [11], [12], which were proposed to play a crucial role in the simulation process [58]. In addition, some authors proposed hybrid approaches, in which the immediate and involuntary understanding of others’ emotions is subserved by mirror mechanisms and embodied simulation, while more sophisticated intersubjective understanding is guided by TT principles [40].

Studies investigating the neural basis of face processing emphasised that especially the processing of the variant aspects of faces, including their emotional expressions, involves the superior temporal sulcus (STS [59]–[62]). STS responses to the image of a face were shown to depend on the immediate perceptual history of that face image and were proposed to play a role in action prediction [63], [64]. The STS may therefore be implicated in the bias in perceptual report found in the current study. However, the presentation of an expressive face triggers not only a pictorial description, it also triggers an intuitive and immediate understanding of the emotional/mental state of the agent. Although the STS may generate expectations, or anticipations, of impending behaviour of others [63], [64] and may play a role in processing others’ intentions [65]–[67], intuitive and immediate understanding is unlikely to be accomplished by just the STS. It has been argued that this would require the recruitment of the motor representation of the observed action and its associated vicero-motor areas, enabled by mirror neuron mechanisms [12]. The STS is extensively connected to mirror neuron areas [70] and substantial evidence has accumulated that the perception of facial expressions indeed activates mirror neuron mechanisms [8], [69]–[72]. Thus, a distributed system, involving the STS and mirror neuron mechanisms, might underlie the tacit/intuitive understanding of others’ facial expressions. This might (top-down) shape the perception of the visual stimulus, without requiring conceptual reasoning [73].

Thus, even though the understanding of other’s facial expressions through embodied simulation mechanisms is considered a bottom-up process, the *anticipated* emotional state that it generates in turn affects top-down the perception of the facial expression. We speculate that this mechanism underpinned the bias in perceptual report of the neutral facial expressions presented at the end of the clips in the current experiments.

### Future Studies

At present, the emotional anticipation hypothesis as explanation for the overshoot response bias is speculative. Even though the experiments show that a bottom-up explanation based on contrast effects, adaptation, and RM, is unlikely, they do not provide direct evidence for a top-down emotional anticipation account. It might be possible to further test this account using deceptive facial expressions [73], which, despite their similarities with genuine expressions in terms of features and geometries, do not reflect the same emotional state of mind as genuine expressions. Future experiments could also use electromyography of the observer’s facial muscles [74] to investigate a possible contribution of embodied simulation. Further investigations could also explore the contribution of a tacit version of TT to emotional anticipation. The involuntary nature of the processes does not need to constitute an obstacle for TT as folk psychological theory is partly tacit [75]–[77].

### Conclusion

The current study showed that distortions in the perception of facial expressions not necessarily arise from low-level visual mechanisms, such as contrast effects, adaptation and RM. We speculated that the perception of an agent’s dynamic facial expression, provided its dynamicity is ecologically-valid, is shaped (distorted) by the involuntary anticipation of the agent’s most likely emotional state of mind in the immediate future. The findings are in line with the recently proposed interactive model of social perception [4] and with the notion of ‘low-level mindreading’ [40], [77]. We speculated that emotional anticipation may be underpinned by mirror mechanisms and embodied simulation [13], [57].
